# Can Seasons be an Etiologic Factor for Monosymptomatic Nocturnal Enuresis?

**DOI:** 10.7759/cureus.2580

**Published:** 2018-05-04

**Authors:** Aykut Bugra Senturk, Cemil Aydın, Musa Ekici, Basri Cakiroglu, Mustafa Sungur, Murat Baykam

**Affiliations:** 1 Urology, Hitit University Corum Erol Olcok Training and Research Hospital, Corum, TUR; 2 Urology, Hisar Intercontinental Hospital, Istanbul, TUR

**Keywords:** children, nocturnal enuresis, seasonal

## Abstract

Introduction

Primary nocturnal enuresis is the most frequent urinary system complaint among pediatric patients.

Material and Methods

Data compiled from 5,500 children, aged between five to 16 years, diagnosed with enuresis during the period from January 2010 to December 2015 were analyzed. The inclusion criteria were having a diagnosis of monosymptomatic nocturnal enuresis, a birth date known for certain, and complete family history taken. A total of 3,547 children met the inclusion criteria and were included in the study. The study was performed by retrospective analyses.

Results

Analysis of the results revealed a statistically significant difference among the rates of enuresis with respect to months and seasons (p < 0.001). In our study, we retrospectively reevaluated 3,500 patients for their birth dates and determined a statistically significant difference in the rates of nocturnal enuresis with respect to seasons (p < 0.001).

Conclusion

As a result of this study, we determined that monosymptomatic nocturnal enuresis in children is more frequent, particularly in those born during the summer months.

## Introduction

Primary nocturnal enuresis is the most common urological complaint in the pediatric population. The disease incidence in 7-year-old patients is 5 - 10%, which drops to 1 - 2% at the age of 15 years old. The annual spontaneous recovery rate is around 15% [1]. Despite the potential for recovery, seven of every 100 children suffer from bedwetting into adulthood. Primary nocturnal enuresis is a health problem that affects the physical, emotional, and social lives of both the patient and his/her family [2].

The International Children’s Continence Society describes four nocturnal enuresis subtypes: monosymptomatic, non-monosymptomatic, primary, and secondary [3]. Bedwetting is accepted as the only symptom of nocturnal enuresis in children older than five years of age. Despite its common occurrence, the pathology and the etiology of this disease are not clear. The most popular opinion suggests that monosymptomatic nocturnal enuresis is a multifactorial disorder, with a combination of somatic, genetic, and behavioral factors. A high volume of urine produced during the night, decreased nocturnal bladder capacity, increased detrusor activity, and arousal disorders also contribute to this multifactorial disorder. Moreover, monosymptomatic nocturnal enuresis is a genetically complex problem, and associated genetic locations have been determined on chromosomes 12, 13, and 22 [2, 4]. In addition, one previous study concluded that seasonal temperature changes might be involved in the etiology of enuresis [5]. 

However, to the best of our knowledge, the frequency of monosymptomatic nocturnal enuresis based on the birth month of the patient has not been evaluated previously. In this study, we retrospectively analyzed the prevalence of monosymptomatic nocturnal enuresis based on the month (January – December) and season (winter, spring, summer, and fall) of the year in which the patient was born.

## Materials and methods

We reviewed patient records from the Hitit University Corum Erol Olcok Training and Research Hospital, Corum, Turkey for our study. The data compiled from 5,500 children between five and 18 years old who lived in Turkey and were diagnosed with enuresis from January 2010 to December 2015 were analyzed. This study was conducted retrospectively in accordance with the principles of the Declaration of Helsinki.

Those patients with bladder dysfunction due to neurological disorders, daytime voiding symptoms, and polyuria due to systemic disorders were excluded from the study. The inclusion criteria were as follows: a diagnosis of monosymptomatic nocturnal enuresis, birth date known with certainty, and complete family history documentation. A total of 3,547 children who met these criteria were included in the study. This study was performed using retrospective analyses. For this research, we aimed to explore whether the birth date was involved in the etiology of monosymptomatic enuresis and to evaluate the effects of the birth month and season on the pathophysiology of nocturnal enuresis if such an etiological factor exists.

The statistical analyses were performed by using the R software package (version 3.2.2, available from https://cran.r-project.org). The descriptive statistics used for the continuous variables were expressed as the mean ± standard deviation. For the categorical data, they were expressed as the number and percent. The multiple group comparisons were tested using the chi-squared test for proportions. The level of significance was set at p < 0.05.

## Results

The mean age of the patients included in this study was 12.08 ± 6.23 years old. Of the patient population, 1,535 were females, and 2,012 were males. The family history of nocturnal enuresis rate in our study was 35% (Table 1).

**Table 1:** Frequency Distribution N: number; Min: minimum; Max: maximum

Nocturnal Enuresis	N	Min	Max	Mean	Standard Deviation
Month	12	199	377	295.58	52.569
Season	4	798	1,013	886.75	91.124

The numbers of patients born in each month were as follows: January- 377 (10.63%), February- 222 (6.26%), March- 272 (7.67%), April- 282 (7.95%), May- 300 (8.46%), June- 312 (8.80%), July- 353 (9.95%), August- 348 (9.81%), September- 313 (8.82%), October- 311 (8.77%), November- 258 (7.27%), and December- 199 (5.61%). A statistically significant difference was found between the enuresis rates with respect to the birth month (p < 0.001) (Table 2).

**Table 2:** Results of Proportion Test by Months N: number; P: p value

	Groups	N	Rate (%)	P
Nocturnal Enuresis	January	377	10.63	< 0.001
	February	222	6.26	
	March	272	7.67	
	April	282	7.95	
	May	300	8.46	
	June	312	8.80	
	July	353	9.95	
	August	348	9.81	
	September	313	8.82	
	October	311	8.77	
	November	258	7.27	
	December	199	5.61	

When the enuresis rates were calculated based on the season, the averages were 24.8% for winter, 28.56% for summer, 24.87% for fall, and 22.5% for spring. A statistically significant difference was found among the nocturnal enuresis rates with respect to the season of the year (p < 0.001) (Table 3).

**Table 3:** Results of Test for Proportion by Season N: number; P: p value

	Groups	N	Rate (%)	P
Nocturnal Enuresis	Spring	854	24.08	< 0.001
	Summer	1013	28.56	
	Fall	882	24.87	
	Winter	798	22.50	

## Discussion

Nocturnal enuresis is a common health problem among six-year-old children in whom it occurs at a rate of 15%. This distressing condition can negatively affect the behavior of both the child and his/her family. The etiology of monosymptomatic nocturnal enuresis is not clearly understood, and it is defined as one symptom developing from a combination of various factors. Good daytime bladder control in children usually develops around two to three years old, in contrast to nighttime bladder control, which develops around three to seven years old [6].

According to the literature, less than one-half of incontinence cases are actually monosymptomatic nocturnal enuresis [7]. Without any urological symptoms, night bedwetting is its only symptom. These patients do not show any other lower urinary tract symptoms, such as daytime incontinence. A high volume of urine produced during the night, decreased plasma vasopressin, uninhibited bladder contractions during the night, and sleep arousal problems are the factors responsible for this multifactorial condition.

A study by Carman et al. reported that the percentage of enuretic cases with a family member with a history of bedwetting was 55.9%. The rate for the enuresis cases without a family member with a history of bedwetting was obviously lower (15.4%), which was highly statistically significant [8]. The family history of bedwetting rate in our study was compatible with that in the literature.

Nocturnal enuresis is a common problem in childhood and adolescence, with prevalence rates of 15 - 20% at five years old, 5% at six to 10 years old, 2 - 3% at 10 - 17 years old, and 1% over 17 years old. The annual spontaneous recovery rate is 15%, and boys suffer from this condition 1.5 times more often than girls [9]. In our study, the ratio of boys to girls was 3:1 (2,012/1,535), which is compatible with the rate stated in the literature. An extensive study from Turkey consisting of 5,522 children reported that the overall prevalence of nocturnal enuresis was 11.5% [10].

Extensive research on various concepts involving the urodynamic, behavioral, genetic, hereditary, hormonal, neurological and physiological conditions, and sleep disorders alone has failed to explain the pathophysiology of monosymptomatic nocturnal enuresis [5]. Genetic factors play an important role in this multifactorial disorder; therefore, patients with nocturnal enuresis usually have a family history of nocturnal bedwetting [2]. In addition, the number of siblings, birth order, the economic and educational status of the family, male sex, and a history of urinary tract infections have also been reported to be related to nocturnal enuresis [11]. In a study exploring the role of environmental factors in the etiology of monosymptomatic enuresis, the winter season was described as a predisposing factor [5].

In our study, we retrospectively reevaluated the birth dates of 3,547 patients and found a statistically significant difference in the nocturnal enuresis rates with respect to the season of the year in which the patient was born (p < 0.001). We observed that monosymptomatic nocturnal enuresis was more common in the patients who were born in the summer months. We also found that the nocturnal enuresis rates were statistically significantly different with regard to the birth month (p < 0.001). In particular, monosymptomatic nocturnal enuresis was more common among those patients born in July, August, and January. A summer birth month was determined to play a significant role in the development of monosymptomatic nocturnal enuresis. However, more comprehensive research must be performed to clearly state the role of the birth month in the etiology of monosymptomatic nocturnal enuresis.

## Conclusions

In this study, we determined that monosymptomatic nocturnal enuresis in children is more common in those who were born during the summer months.
